# Inter-observer agreement of Medical Research Council-sum score and handgrip strength in the ICU

**DOI:** 10.1186/cc9949

**Published:** 2011-03-11

**Authors:** G Hermans, B Clerckx, T Vanhullebusch, J Segers, G Vanpee, C Robbeets, M Casaer, P Wouters, R Gosselink, G Vandenberghe

**Affiliations:** 1 UZ Leuven, Belgium

## Introduction

Muscle weakness often complicates critical illness and is associated with prolonged duration of rehabilitation, increased mortality and limiting functional outcome even years later [1]. To examine the effects of interventions on this complication, reliable measurements of muscle force in critically ill patients are needed. We aimed to examine, in critically ill patients, the inter-observer agreement on the Medical Research Council (MRC)-sum score and handgrip strength, two methods to quantify muscle force.

## Methods

All patients were included in an ongoing randomized controlled trial examining two feeding strategies (ClinicalTrials. gov:NCT 00512122) and constituted a cross-sectional, randomly selected sample. Patients were studied on median ICU day 17 (11 to 29.5). Two observers independently measured the MRC-sum score in 75, and handgrip strength in 46 critically ill patients.

## Results

The intra-class correlation coefficient (ICC) for the MRC-sum score was 0.95 (0.92 to 0.97), with weighted kappa coefficient for individual muscle group scores of 0.83 ± 0.03. Kappa coefficient was 0.68 ± 0.09 for identifying patients with MRC-sum score <48, 0.88 ± 0.07 for MRC-subtotal in the upper limbs <24, and 0.93 ± 0.07 for MRC-sum score <36. The ICCs for left and right handgrip strength were respectively 0.97 (0.94 to 0.98) and 0.93 (0.86 to 0.97).

## Conclusions

We found very good inter-observer agreement, both for MRC-sum score and for handgrip strength in critically ill patients. When applying MRC-sum score <36 as a cut-off for severe weakness, agreement was excellent supporting its use as an outcome parameter for interventional studies. Agreement on identifying significant weakness (MRC-sum <48) was good. For an equivalent cut-off to identify significant weakness in the upper limbs (< 24), agreement was very good. It remains to be determined whether this may be used as a substitute for the total MRC-sum score.
